# Hemophagocytic lymphohistiocytosis caused by multiple infections during primary chemotherapy for pediatric acute lymphoblastic leukemia: a case report

**DOI:** 10.3389/fimmu.2024.1438378

**Published:** 2024-11-06

**Authors:** Yaning Ao, Yusheng Huang, Xiaobo Zhou, Jiawen Li, Qing Zhang, Sujun Wu, Ying Fu, Jinfeng Zhang

**Affiliations:** ^1^ Department of Neonatology, Shunde Women and Children’s Hospital of Guangdong Medical University, Foshan, China; ^2^ Institute of Maternal and Child Research, Shunde Women and Children’s Hospital of Guangdong Medical University, Foshan, China; ^3^ Department of Hematology-Oncology, Shunde Women and Children’s Hospital (Maternity and Child Healthcare Hospital of Shunde Foshan), Guangdong Medical University, Foshan, China; ^4^ Department of Obstetrics, Shunde Women and Children’s Hospital (Maternity and Child Healthcare Hospital of Shunde Foshan), Guangdong Medical University, Foshan, China

**Keywords:** hemophagocytic lymphohistiocytosis, pediatric acute lymphoblastic leukemia, multiple infections, *UNC13D*, ruxolitinib

## Abstract

Hemophagocytic lymphohistiocytosis (HLH) is a life-threatening hyperinflammatory disorder that occurs as a consequence of immune dysregulation. HLH can be primary (familial or non-familial) or secondary to infection, autoimmune disease or malignancy. Malignancy-associated HLH is often accompanied by hematologic and lymphoid neoplasms. This report describes the case of a 3-year-old girl with an initial diagnosis of acute lymphoblastic leukemia who subsequently developed HLH during primary chemotherapy. She was admitted with a pulmonary infection, and initial blood tests showed thrombocytopenia and anemia. Whole-exome sequencing of gene and whole transcriptome RNA sequencing data indicated mutations of *UNC13D*. The hospital course was complicated by multiple infections, altered mental status and acute respiratory distress syndrome. HLH secondary to multiple infections that achieved remission following targeted therapy with ruxolitinib, in conjunction with corticosteroids and other complementary treatments. This report provides a synopsis of the diagnostic and treatment procedures implemented in this case.

## 1 Introduction

Hemophagocytic lymphohistiocytosis (HLH) is a life-threatening hyperinflammatory disorder that is caused by immune dysregulation. HLH is diagnosed using the standard molecular or clinicopathologic criteria proposed by Henter et al. in 2004 (**Supplementary Table 1**), whereby five out of eight points need to be met (1). HLH may be primary (familial or non-familial) or secondary. Primary HLH is often observed in childhood in association with a number of genetically heterogeneous disorders and is typically triggered by common infections. Secondary HLH is typically linked to an infection, malignancy, or autoimmune disease, without any explicit genetic predisposition toward HLH. In instances where HLH or a syndrome mimicking HLH transpires in the context of a rheumatologic or autoinflammatory disease, it is frequently termed as macrophage activation syndrome (MAS) (2). Therein, malignancy-associated HLH may be triggered by malignancy or by chemotherapy and is typically associated with lymphoid neoplasms, leukemias and occasionally with solid tumors. In chemotherapy-associated HLH, the initiating triggers include direct cytotoxic effects, secondary infection, or the hyperinflammatory state caused by the treatment (3).

The patient described in this report was a 3-year-old girl who was diagnosed initially with acute lymphoblastic leukemia and subsequently developed HLH following primary chemotherapy. The condition of HLH demonstrated improvement following a targeted treatment with ruxolitinib, in conjunction with corticosteroids, several broad-spectrum antimicrobials, tracheal intubation, ventilator-assisted ventilation, and immunoglobulin therapy. This report summarizes the diagnostic process and treatments used in this case.

## 2 Case presentation

The patient was a 3-year-old girl who was hospitalized with fever and cough. Routine blood tests at a local clinic suggested leukocytosis, moderate anemia, and thrombocytopenia. She was then transferred to our hospital, where a bone marrow puncture was performed. The initial bone marrow examination shows that both primary lymphocytes and naive lymphocytes are at 52%, and the minimal residual disease (MRD) shows that abnormal naive B lymphocytes accounted for 61.9% (**Supplementary Table 2**).The results of initial bone marrow examination,MRD and immunophenotyping studies indicated acute B-cell lymphoblastic leukemia (common B-ALL).

The VDLD (vincristine 1.5mg/m^2^, daunorubicin 30mg/m^2^, L-asparaginase 2500U/m^2^, and dexamethasone 6mg/m^2^) chemotherapy regimen was started on day 8 after admission. Two administrations of intrathecal injection, comprising cytarabine (30mg), methotrexate (12mg), and dexamethasone (5mg), were executed as a prophylactic measure against central leukemia. The patient experienced recurrent fever during the hospital course. Her body temperature fluctuated between 37°C and 39°C but gradually increased, peaking at 39.5°C. The cerebrospinal fluid cultures and cytology were negative. Blood culture results suggested *C. tropicalis* infection. Blood metagenomic next-generation sequencing revealed 104 specific reads of human herpes virus type 5 and 239 specific reads of *C. tropicalis*. High-throughput sequencing of bronchoscopic alveolar lavage fluid also identified *C. tropicalis*, *P. jirovecii*, and human metapneumovirus (**Table 1**). The third vincristine and daunorubicin chemotherapy were discontinued in view of severe infections, and dexamethasone treatment continued. The fever persisted despite treatment with broad-spectrum antibacterial including vancomycin, meropenem, imipenem, tigecycline and oral trimethoprim/sulfamethoxazole (TMP/SMX), antifungal (caspofungin and fluconazole), and antiviral (ganciclovir) therapy.

**Table 1:** Summary of the pathogene findings in our patient.

Pathogene	Sample	Method	The number of sequence
Human herpesvirus 5	Blood	NGS	104
Candida tropicalis	Blood	NGS	239
	BALF	NGS	37804
Pneumocystis jirovecii	BALF	NGS	947
Human metapneumovirus	BALF	NGS	12415

NGS, next-generation sequencing; BALF, bronchoscopic alveolar lavage fluid.

**Figure 1:**
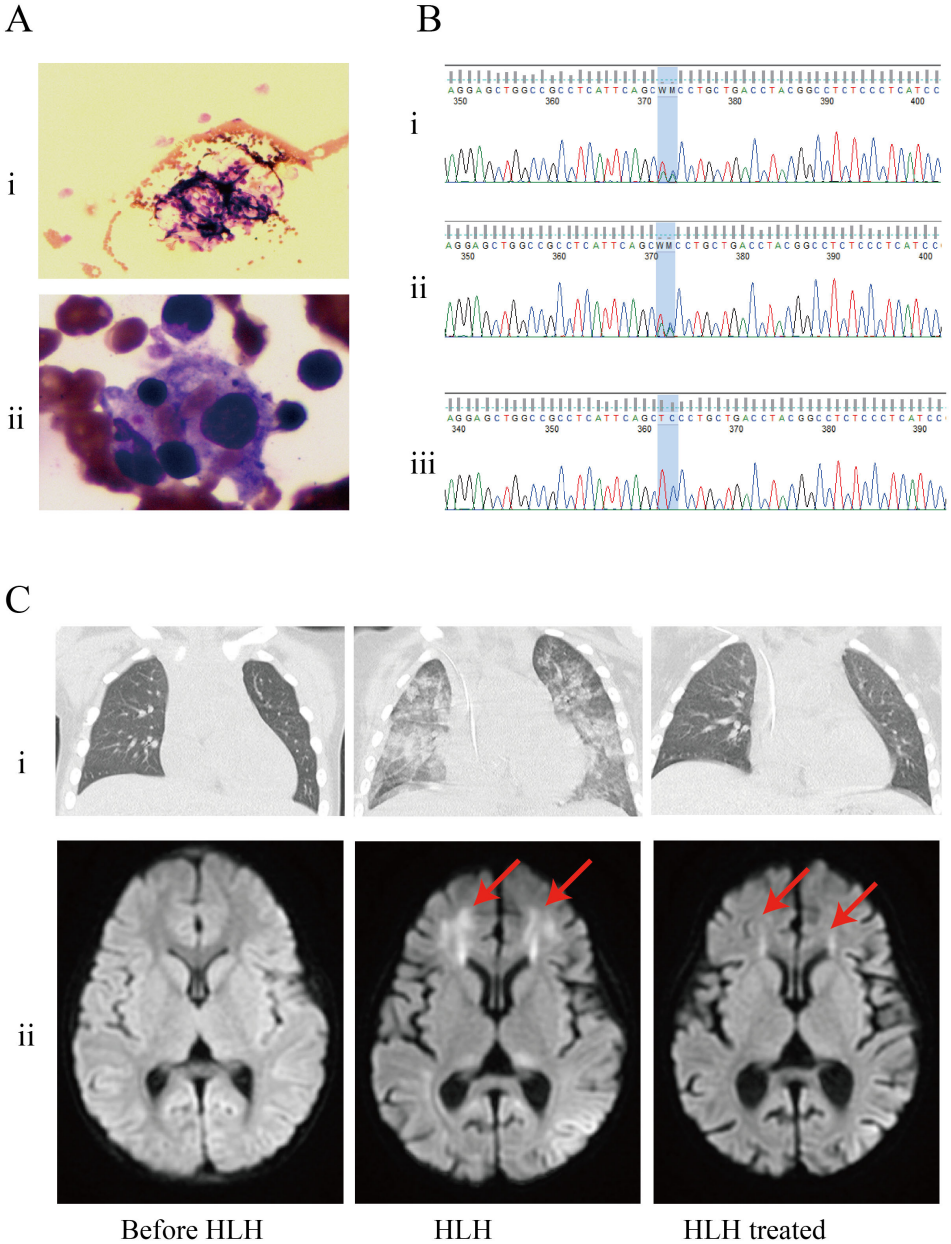
Patients examination results. **(A)** Bone marrow biopsy found histiocytes phagocytosing blood cells. (i) Histiocytes phagocytosed blood cells (arrow); (ii) Hemophagocytic cells underwent phagocytosis (arrow). **(B)** Results of whole-exome sequencing: the genetic analysis identified that the patient and her father had heterozygous mutations in UNC13D. (i) patient; (ii) father; (iii) mother. **(C)** The results of imaging examinations were as follows, (i) CT scan showed exudative lesions in both lungs; (ii) brain magnetic resonance imaging (MRI): a T2−weighted sequence showing abnormal white matter signal (arrow).

One month after admission, she developed tachypnea of sudden onset with tachycardia, altered mental status, and cyanosis with an increase in temperature to 40°C. Physical examination revealed hepatosplenomegaly (liver 3 cm, spleen 1.5 cm below the costal margin). Blood tests revealed severe trilineage cytopenia with neutropenia. Soluble CD25 and ferritin levels were increased (sCD25, 10,065 U/L; ferritin, 4070 µg/L). Flow cytometry showed decreased natural killer (NK) cell activity (**Table 2**). Bone marrow biopsy revealed histiocytes with hemophagocytic activity (**Figure 1A**). Whole-exome gene sequencing data for the patient and her father indicated mutations of *UNC13D* (**Figure 1B**). Specifically as follows: the patient shows a mutation at position 370, where the normal base sequence is altered. The shaded area indicates the mutation position, which likely represents a heterozygous mutation (**Figure 1B**i). The father’s sequencing also shows the same variant in the same position (highlighted area), suggesting that he may be a carrier of this mutation (**Figure 1B**ii). The mother’s sequencing does not appear to show the mutation at this position, implying that she does not carry this particular mutation in the *UNC13D* gene (**Figure 1B**iii). The site information regarding the *UNC13D* gene mutation is as follows:1) The *UNC13D* gene mutation site information from her father as shown in **Supplementary Table 3**: UNC13D: NM_199242.2:c.1201_1202delinsAA (p.Ser401Asn); 2) The site information of UNC13D gene mutation of the patient included the result of whole-exome sequencing indicated that ‘c.1201_1202delinsAA p.Ser401Asn’ in **Supplementary Table 4**.

**Table 2:** Summary of the laboratory findings in the patient.

	Unit	Normal Range	Before HLH	HLH	HLH-treated
Ferritin	ng/ml	13-150	933	4070	2090
Triglycerides	mmol/L	0.56-1.7	1.01	3.69	1.66
Fibrinogen	g/L	2-4	1.49	0.36	1.66
NK cell activity	%	≥15.11	NA	15.20	14.92
Souble CD25	U/ml	223-710	NA	10065	1121
Hemoglobin	g/L	≥110	77	49	106
Platelets	*10^9/L	100-300	8	1	207
WBC	*10^9/L	4.4-11.9	0.91	0.16	5.77
Neutrophils	*10^9/L	1.2-7	0.18	0.00	3.03
IL-6	pg/ml	≤5.04	NA	219.89	2.61
IL-10	pg/ml	≤5.00	NA	13.84	4.24
G test	pg/ml	NA	>600	331	178.55

HLH, Hemophagocytic lymphohistiocytosis; NK, natural killer; WBC, white blood cell; IL, Interleukin; NA, No available; G test, 1,3-β-D-glucan test.

Besides, whole transcriptome RNA sequencing was also performed on the patient. The result of whole transcriptome RNA sequencing indicated that ‘NM_199242.3:exon14:c.1201_1202 delinsAA:p.S401N’ in **Supplementary Table 5**. Next, we also detected expression of HLH-related markers such as CD107a, the degranulation function of which was decreased in NK cells (ΔCD107a 9%, normal >10%) and normal in cytotoxic T lymphocytes (ΔMFI 4.8%, normal ≥2.8%). CT scan showed exudative lesions in both lungs and brain magnetic resonance showed white matter lesions (**Figure 1C**). The patient was diagnosed with HLH based on the HLH-2004 diagnostic criteria. Endotracheal intubation and mechanical ventilation were initiated to manage respiratory distress. Intravenous methylprednisolone (initial 2mg/kg, peak at 10 mg/kg), immunoglobulin, intravenous TMP/SMX (0.18g per dose, twice a day) and ruxolitinib (5mg, every12h) were administered daily for 8 days. During this period, dexamethasone was tapered, and discontinuated in 3 days. Her fever, altered mental status and dyspnea improved, and her HLH biomarker levels gradually decreased (sCD25 to 1121 U/L; ferritin to 2090 µg/L) (**Table 2**).

**Figure 2:**
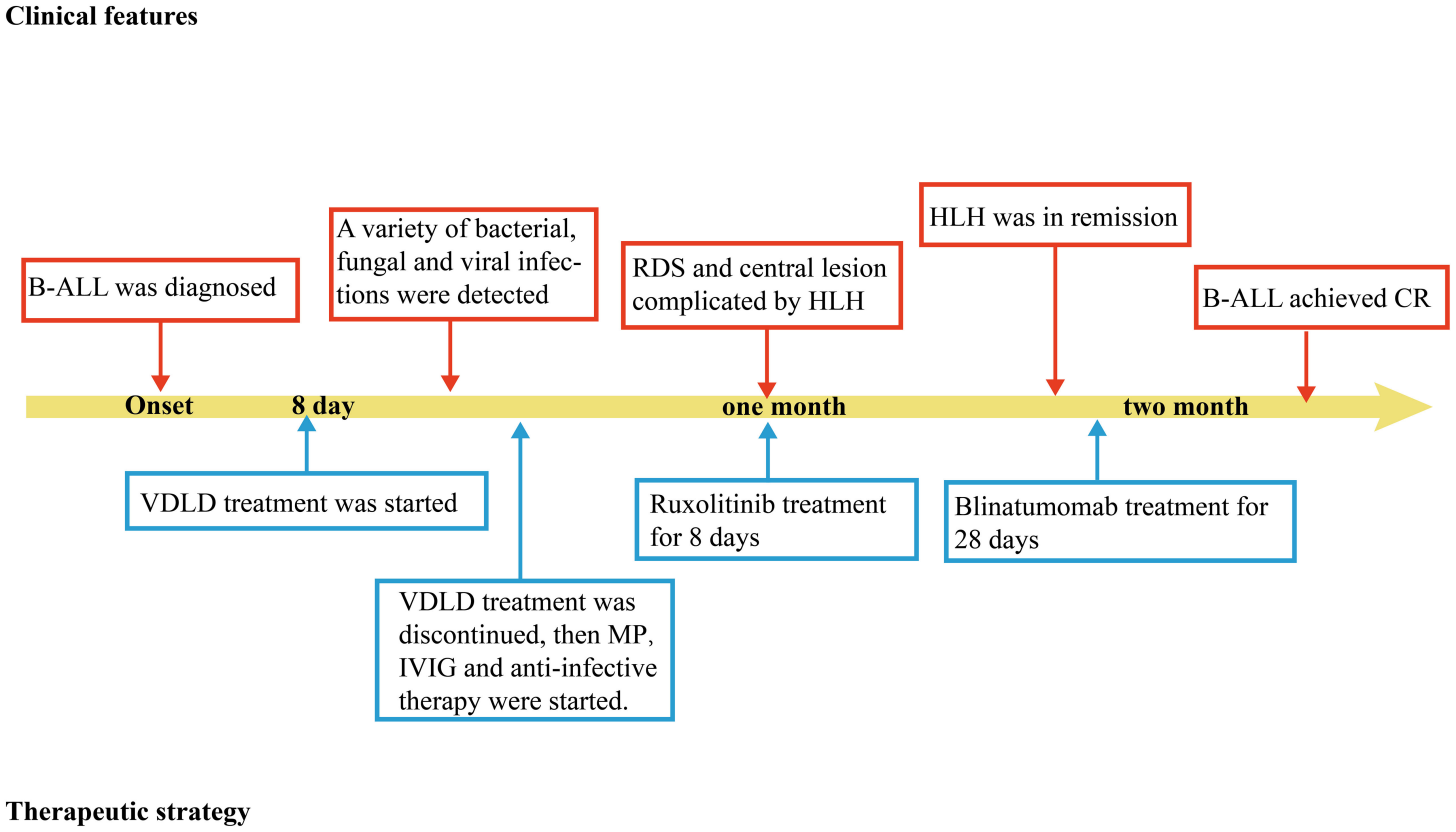
Timeline depicting the disease course of the patient. The timeline illustrates the different events in the course of the patient’s treatment and disease progression. MP, methylprednisolone; B-ALL, B-cell acute lymphoblastic leukemia; IVIG, intravenous immunogloblin; RDS, respiratory distress syndrome. HLH, Hemophagocytic lymphohistiocytosis; CR, complete remission.

When the patient’s condition had stabilized, she received the third intrathecal injection and bridging chemotherapy (blinatumomab for 28 days) for her B-ALL and the follow-up bone marrow examination shows that both primary lymphocytes and naive lymphocytes are at 0%, and the follow-up MRD is negative, indicating that the patient has achieved complete remission (CR) (**Supplementary Table 2**). She eventually improved and remained stable. The timeline in **Figure 2** includes key events throughout the disease course of the patient.

## 3 Discussion

HLH is a syndrome describing patients with severe systemic hyper-inflammation, caused by immune dysregulation with abnormal or impaired function of NK cells and cytotoxic T lymphocytes and characterized by fever, pancytopenia, splenomegaly, and hemophagocytosis in the bone marrow, liver, and lymph nodes. Critical HLH patients can also develop rash, hepatitis, disseminated intravascular coagulation, acute liver failure, central nervous system (CNS) involvement, multiorgan failure and death. The high mortality rate makes prompt recognition and treatment of this hyperinflammatory syndrome essential (2).

Survival rates of 56% following the acute phase and 36% at 5 years have been reported for pediatric patients with HLH (4). Infection, malignancy (particularly lymphoma), autoimmune disease, and genetic defects are known to be common etiologies of pediatric HLH (5). According to the latest consensus recommendations, HLH associated with malignancy can manifest initially as a consequence of the malignant disease, referred to as malignancy-triggered HLH, or can occur in the context of myelosuppression induced by chemotherapy, known as chemotherapy-associated HLH. Malignancy-triggered HLH occurs most frequently in patients with T-cell or NK-cell lymphoma or leukemia, diffuse large B-cell lymphoma, or Hodgkin lymphoma (6). Reports on B-ALL-related HLH are rare. Martinez-Romera et al. reported two cases of HLH that developed during the maintenance phase of chemotherapy for ALL (7). In 2008, O’Brien et al. reported nine cases of B-ALL-associated HLH, four of which were fatal (8). Our report describes a rare case of B-ALL-associated HLH during primary chemotherapy. The patient achieved CR in HLH following treatment with ruxolitinib and methylprednisolone.

Owing to a paucity of clinical trials, the recommended management for HLH includes steroids (dexamethasone), immunosuppression (e.g., etoposide or ciclosporin), and intrathecal therapy (e.g., methotrexate or steroids). Rituximab might be beneficial for Epstein–Barr virus-related HLH. Additional therapies including alemtuzumab, anti-thymocyte globulin, interferon-gamma blockers, and salvage therapy are also used to treat HLH (9). Treatment varies according to type of HLH. For primary HLH, the HLH-94 and HLH-2004 protocols are mostly used (10, 11). Notably, a cure can only be obtained via allogeneic hematopoietic stem cell transplantation, in which the defective immune system is replaced with a healthy one (12). Treatment of secondary HLH always starts with targeting of the underlying trigger using agents to combat infection, malignancy, or autoimmune disease. However, in some cases, it may also be necessary to treat the associated hyperinflammation. Various agents have been used to treat hyperinflammation, including dexamethasone and, in the most severe cases, etoposide (13). Agents that block the effects of individual cytokine have also been employed. This approach is particularly appealing for patients in whom HLH is triggered by infection and administration of cytotoxic or other immunosuppressive agents that could compromise clearance of the inciting pathogen (14, 15). Despite use of currently available therapies, many patients with HLH succumb to the disease. To improve HLH patient outcomes, studies have focused on the use of novel cytokine-directed therapies to dampen hyperinflammation.

Many of the cytokines that are elevated in HLH, including interferon-gamma, interleukin (IL)-2, IL-6, IL-10, IL-12, and granulocyte macrophage-colony stimulating factor, signal through a pathway that involves the Janus kinases and signal transducers and activators of transcription. Ruxolitinib, as a strong Janus kinase inhibitor, can exert an anti-hyperinflammatory effect in HLH by blocking multiple cytokines. Compared with intensive chemotherapy, ruxolitinib has low toxicity and is well tolerated (16). Zhang et al. used ruxolitinib to treat 12 pediatric patients with newly diagnosed secondary HLH. Ten patients experienced a favorable response at 28 days, with eight exhibiting a CR. Among these eight, seven maintained the CR for more than 6 months (17). In 2022, Zhang et al. continued to treat 52 pediatric HLH patients with ruxolitinib monotherapy, the results indicated that 22 patients achieving CR, 9 patients achieving partial remission (PR), and 5 patients showing HLH improvement. Notably, 3 out of the 4 primary HLH patients achieved CR after ruxolitinib monotherapy (18). These results indicated the potential effectiveness of ruxolitinib as a first-line treatment for pediatric HLH. Nonetheless, the ideal dosage and timing of ruxolitinib administration still require definitive determination. Furthermore, there is a need for comprehensive studies that investigate treatment plans incorporating ruxolitinib and other HLH-targeted agents for infants and young children diagnosed with HLH. These studies are essential to gain a more profound understanding of the efficacy and safety of ruxolitinib in such contexts.

It is worth noting that the patient developed transient altered mental status when her HLH was at its most severe. Craniocerebral magnetic resonance scans obtained at this time indicated acute White matter injury. Following the administration of ruxolitinib for targeted treatment of HLH, the patient regained normal consciousness. Significant absorption of the white matter lesion was evident in the subsequent plain magnetic resonance imaging. The patient was diagnosed with CNS involvement secondary to HLH (CNS-HLH) according to the latest diagnostic criteria. CNS-HLH can be familial, non-familial, or isolated. The severity of CNS-HLH varies widely but usually heralds a poor prognosis, and treatment of refractory CNS-HLH is challenging (19). The standard treatment for CNS-HLH is a combination of methotrexate and dexamethasone intrathecal injection (20). Nonetheless, the majority of patients suffering from CNS-HLH are not suitable candidates for intrathecal injection when their condition is critical. Furthermore, the CNS-HLH can potentially benefit from the HLH-94 and HLH-2004 protocols, or from innovative cytokine-directed therapies specifically designed to target HLH (21). There has been a case report suggesting that Lazarus is effective for non-familial CNS-HLH (9). Indeed, the absence of a specifically developed pharmaceutical treatment for CNS-HLH significantly complicates the management of this condition.

Previous studies demonstrated that ruxolitinib reversed CNS inflammation and prolonged survival in murine models of HLH (22). Results of another animal experiments indicated that ruxolitinib could penetrate the blood brain barrier of mice and reduce CNS involvement in the Rab27a-/- mice (an animal model of HLH) (23). However, these findings haven’t been confirmed in human patients. Ge J et al. considered that ruxolitinib is probably not an ideal drug for CNS involvement in primary HLH according their latest clinical research results. In this study, all pediatric HLH patients with CNS involvement received intrathecal therapy and ruxolitinib treatment. The results indicated two patients with CNS-HLH died, two patients achieved PR and four patients with CNS involvement relapsed after achieving CR (24). In fact, CNS symptoms have been regarded as a predictor of mortality in children with HLH (25). Firstly, their subjects were primary HLH patients with CNS symptoms, who had a high likelihood of progression to refractory or recurrent HLH and a poor clinical prognosis in the research conducted by Ge J. Seconldy, these CNS-HLH patients were also administered intrathecal injections, which is considered the standard treatment for CNS-HLH. Despite this, there was no significant enhancement in the patients’ prognosis, suggesting that the treatment of these CNS-HLH patients is extremely difficult. Thus, we do not consider this result sufficient to indicate that ruxolitinib may not be effective for treating CNS-HLH. In our case, the patient’s CNS symptoms were attributed to acute neuroinflammatory caused by HLH. Ruxolitinib delivered across the Blood-Brain Barrier to exert the potent anti-inflammatory effect as a potential mechanism for ameliorating the CNS-HLH The effectiveness of ruxolitinib in treating refractory HLH necessitates further empirical evidence, particularly in cases involving CNS, which requires additional investigation.

Anakinra, one of cytokine-directed therapies, is first-line recommended for the treatment of HLH for adults and children in all ages according to the studies from western countries (26, 27). However, anakinra is recommended as grade B in Chinese guidelines for diagnosing and treating HLH due to the limited evidence reported within China. This rationale elucidates why anakinra is not regarded as the preferred therapeutic option for HLH in the current case. Additionally, dexamethasone is recognized as the preferred corticosteroid for HLH treatment, especially in CNS-HLH treatment based on previous research evidence. However, in the present case, we used methylprednisolone instead of dexamethasone for the treatment of CNS-HLH for several reasons: firstly, the chemical structure of methylprednisolone is characterized by the presence of eight hydrogen bonds and three methyl groups, which confer advantageous lipid solubility and facilitate its passage across the blood-brain barrier. Compared to dexamethasone, which contains a fluoride side chain, methylprednisolone exhibits enhanced lipid solubility, potentially resulting in superior permeability through the blood-brain barrier (28); secondly, previous research has demonstrated that dexamethasone, a long-acting glucocorticoid, exhibits potent and prolonged pharmacological effects, including significant immunosuppressive properties. Consequently, its use is associated with an increased risk of secondary severe bacterial infections and opportunistic infections, such as tuberculosis, herpes zoster, and Pneumocystis carinii pneumonia, particularly during the treatment of HLH (29). Methylprednisolone is classified as a medium-acting glucocorticoid with a shorter half-life compared to that of dexamethasone, which indicated its immunosuppressive effect was less pronounced compared to that of dexamethasone. Thus, we consider that methylprednisolone demonstrates a lower propensity for inducing severe adverse effects compared to dexamethasone; Lastly, an modified HLH-04 regimen from Beijing Children’s Hospital involved substituting dexamethasone with methylprednisolone to mitigate the incidence and mortality of chemotherapy-related complications in the treatment of pediatric HLH.The improved protocol reduced the intensity of chemotherapy, with overall efficacy no worse than the standard HLH-04 regimen, and significantly reduced the rate of chemotherapy-related myelosuppression, fungal infection and mortality. The results indicated that there was no statistically significant difference in treatment outcomes following substituting dexamethasone with methylprednisolone for CNS -HLH patients. This lack of significant difference did not impact the long-term prognosis of pediatric HLH with CNS involvement (30). According to the above, comparing to dexamethasone, methylprednisolone can exert comparable effect with fewer severe adverse effects in pediatric CNS-HLH.

Finally, the patient’s genetic test report revealed a heterozygous mutation in the *UNC13D* gene, implying a familial predisposition toward HLH. A total of 12 causative genes were identified between 1999 and 2018 (13). Familial Hemophagocytic Lymphohistiocytosis (FHL) is a rare autosomal recessive disorder caused by mutations in *PRF1* (resulting in FHL2), the UNC13 homologue D gene (*UNC13D*, resulting in FHL3), the synapse fusion protein 11 gene (*STX11*, resulting in FHL4), or synapse fusion protein caused by mutations in the binding protein 2 gene (*STXBP2*, resulting in FHL5) and usually occurs in infancy (31). The latest data indicate that *PRF1* and *UNC13D* mutations are quite common (32–34). According to the ClinVar database, there are currently more than 300 *UNC13D* mutations confirmed to be associated with FHL. Although genetic mutations in the FHL subtype vary, they all lead to impaired function of NK cells and T cells (35). They are correlated in granulose-mediated cytotoxic pathways, interfering with perforin-mediated cytotoxic functions, thereby blocking the mechanism that triggers apoptosis or activates induction of apoptosis. Cytotoxic T cells and NK cells cannot effectively kill target cells, so the immune response is overactivated and the inflammatory state persists, causing HLH. The patient and her father had a simple heterozygous *UNC13D* mutation, and whether this mutation can cause HLH is controversial. In fact, whole transcriptome RNA sequencing data for the patient also indicated mutations of *UNC13D.* Next, We also detected expression of HLH-related markers such as CD107a, the degranulation function of which was decreased in NK cells. Combined with the aforementioned results, the diagnosis of primary HLH could not be completely ruled out. Further functional examination should be conducted on the patient’s father.

## 4 Conclusion

HLH requires early diagnosis and should be considered in patients with ALL who present with pancytopenia. CNS involvement associated with HLH should not be ignored, and magnetic resonance imaging of the brain can identify CNS-HLH early. Infection and inflammation tend to be noticed and treated promptly, but HLH is harder to diagnose in a timely manner because of its rarity and the low likelihood of testing for immune system dysregulation and impaired activation. HLH-related biomarkers need further investigation. Genetic testing should be performed in all patients with HLH. The role of HLH-related heterozygous gene mutations should be emphasized. More prospective evidence is needed regarding the timing, dose, and efficacy of ruxolitinib therapy in HLH and CNS-HLH. Diagnostic vigilance and prompt treatment are essential in the management of HLH.

## Data Availability

The raw data supporting the conclusions of this article will be made available by the authors, without undue reservation.
