# Supplementary material for: Hemophagocytic lymphohistiocytosis caused by multiple infections during primary chemotherapy for pediatric acute lymphoblastic leukemia: a case report
Source: Front Immunol. 2024 Nov 6;15:1438378. doi: 10.3389/fimmu.2024.1438378 (PMC11576205; doi:10.3389/fimmu.2024.1438378)
Supplement: Supplementary file 1 [file Table1.docx]

**Table 1 Diagnostic criteria of HLH adapted from the standard diagnostic criteria proposed in 2004 by Henter et al.**

| **Diagnosis Is Established If One of Either (A) or (B) Is Fulﬁlled** |
| --- |
| (A)Molecular diagnosis consistent with HLH  Pathological mutation : PRF1, UNC13D, STX11, STXBP2, Rab27a, LYST, SH2D1A, BIRC4, ITK, AP3β1, MAGT1, CD27 and so on. |
| (B) Clinicopathologic criteria for HLH fulﬁlled (5 out of the 8 criteria shown below) |
| 1. Fever ≥ 38.5 ℃ for ≥ 7 days |
| 2. Splenomegaly ≥ 3 ﬁnger breadth below the left subcostal margin |
| 3.Cytopenias affecting ≥ 2 of 3 lineages in peripheral blood: Hemoglobin < 90 g/L; Platelets < 100 * 10^9/L;Absolute neutrophil count < 1.0 * 10^9/L |
| 4. Hypertriglyceridemia and/or hypoﬁbrinogenemia: Fasting triglycerides ≥ 265 mg/dL, Fibrinogen ≤1.5 g/L |
| 5. Hemophagocytosis in the bone marrow or spleen or lymph node |
| 6. Low or absent NK cell activity (according to the local laboratory reference) |
| 7. Ferritin ≥ 500 µg/L |
| 8. Soluble CD25 (sIL-2 receptor) ≥ 2400 U/mL |

**Table 2 Summary of the condition of B-ALL in the patient.**

|  | **bone marrow cell morphology** | **MRD** | **Ig-NGS** | **PAX5/CBFA2T3** | **IKZF1** |
| --- | --- | --- | --- | --- | --- |
| **Initial diagnosis** | Primary lymphocyte + naive lymphocyte=52% | Abnormal naive B lymphocytes  61.9% | IgH-SEQ1: 97.88%  IGL-SEQ1：3.06% | (+) | (+) |
| **Before HLH** | Primary lymphocyte + naive lymphocyte＝4.5%  (VDLD induced) | Abnormal naive B lymphocytes  2.4% | NA | (-) | (-) |
| **HLH-treated** | Primary lymphocyte + naive lymphocyte＝0%  (Blinatumomab induced for 28 days) | (-) | 0% | (-) | (-) |

NGS: next-generation sequencing; NA: No available.

| **Gene** | **Position** | **dsSNP_ID** | **Variation** | **Variation type** | **Source** |
| --- | --- | --- | --- | --- | --- |
| UNC13D | Chr17:73832749 | Rs2064911558 | UNC13D: NM_199242.2:c.1201_1202delinsAA (p.Ser401Asn) | Embryos | Father |

**Table 3** **The site information of UNC13D gene mutation of her father.**

**Table 4 The site information of UNC13D gene mutation of patient in whole-exome sequencing data.**

| **Gene** | **Position** | **Variation site** | **Genotype** |
| --- | --- | --- | --- |
| UNC13D NM_199242.2 | chr17 73832749 | c.1201_1202delinsAA p.Ser401Asn | Heterozygosis |

**Table 5 The site information of UNC13D gene mutation of patient in whole transcriptome RNA sequencing data.**

| **Gene** | **Variation** | **Position** | **Frequency** |
| --- | --- | --- | --- |
| UNC13D | NM_199242.3:exon14:c.1201_1202delinsAA:p.S401N | chr17:73832749GA>TT | 50.0% |
